# Response surface data for sensitivity study of industrial spray injected fluidized bed reactor

**DOI:** 10.1016/j.dib.2018.09.105

**Published:** 2018-10-03

**Authors:** Alexander W. Abboud, Donna P. Guillen

**Affiliations:** Materials Science and Engineering Department, Idaho National Laboratory, United States

## Abstract

An industrial fluidized bed reactor was designed to convert an aqueous solid laden stream into a consistent granular product. CFD simulations were run using the MFiX two-fluid model for a fluidizing bed operating at 650 °C. A set of simulations were run over a Latin-hypercube sample of five model parameters – bed particle size, bed particle density, coal particle size, spray feed flow rate, and fluidizing gas flow rate. Data from the simulations were collected on three quantities of interest – bed differential temperature, low solids velocity, and bed void fraction. The data presented here is the full set of response surfaces generated using the process Gaussian response surface model in the Dakota toolkit, as well as the table of data for coefficients of the fitted model. The fits to the five-dimensional Gaussian Process models were 0.7797, 0.8664, and 0.9440 for the temperature, velocity, and solids packing, respectively.

**Specifications table**

**Table:** 

Subject area	*Chemical Engineering*
More specific subject area	*Fluidized Bed Reactor*
Type of data	*Graphs*
How data was acquired	*CFD simulation data*
Data format	*Simulation data fitted with Gaussian Process Regression surfaces*
Experimental factors	*N/A*
Experimental features	*N/A*
Data source location	*N/A*
Data accessibility	*Not public data*
Related research article	*Abboud, A.W., Guillen, D.P. Sensitivity Study of a Full-Scale Industrial Spray-Injected Fluidized Bed Reactor.*[1]

**Value of the data**

• This data examines the results of a fluidized bed reactor utilizing a spray injection system for conversion of liquid material into a solid product.
• This data can provide guidance for similar types of reactors for studying the parameters which affect the fluidization properties.
• The temperature difference seen in this CFD simulations can be compared to other simulations for comparison purposes on the well-mixed behavior of fluidized beds.

## 1 Data

**Fig. 1:**
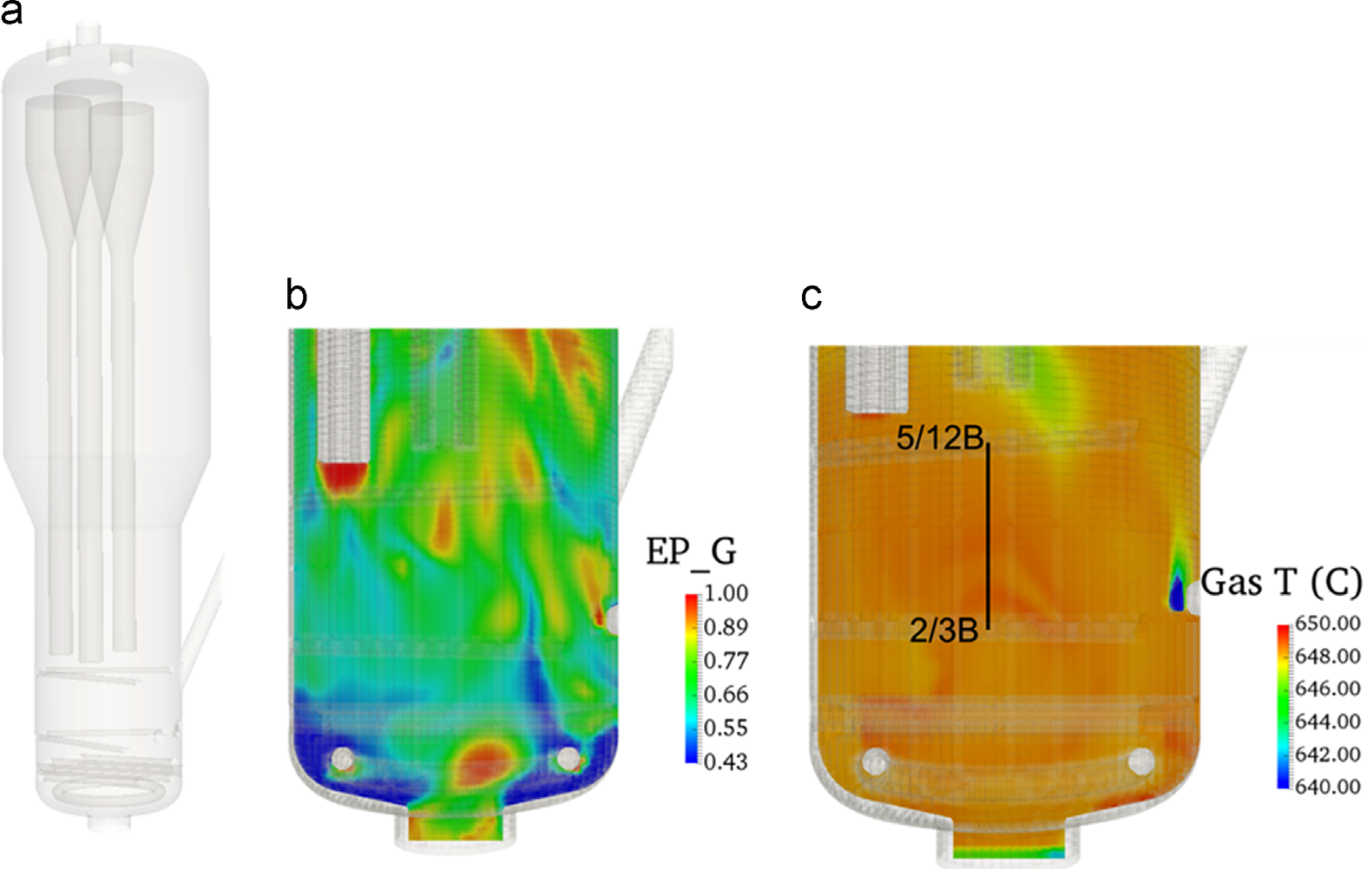
(a) CAD drawing of the FBR, (b) single time setup of void fraction, and (c) gas temperature profile.

A CFD model for a fluidized bed reactor (FBR) was constructed. This FBR is industrial scale with a height of 6.6 m and a width of 1.2192 m. Fig. 1 shows the CAD layout of the FBR, as well as void fraction and temperature differentials in the lower portion of the bed. The off-gas and solids removal portions are clipped from the computational domain, the CFD mesh is approximately 400,000 cells, each simulation took about eight days running on 450 CPUs to simulate 30 s of physical time. The data is time-averaged after reaching a pseudo-steady state condition after 10 s of physical time. The simulations were completed with the MFiX (Multiphase Flow with Interphase eXchanges) code [2] using the Gidaspow drag model [3], with basic evaporation rates used for the aqueous inlet [4], pyrolysis and gasification models for coal [5], water gas shift reactions [4] and one step combustion reactions for the volatile matter [6], [7]. The MFiX code has been used in several fluidized bed validation studies of the model [8], [9], [10]. Further details on the modeling of the full system are found in the full article by Abboud and Guillen [1]. The response surfaces are created using Gaussian process regression models using a five-parameter Latin Hypercube sampling (LHS) [11] in the Dakota (Design Analysis Kit for Optimization and Terascale Applications) toolkit [12].

## 2 Experimental design, materials, and methods

The initial conditions for the FBR are given in Table 1 and the base model parameter conditions are shown in Table 2. For the parameter variation in the LHS, all five of the parameters - bed particle size, bed particle density, coal particle size, spray feed flow rate, and fluidizing gas flow rate – were normalized from 0 to 1 based on the minimum and maximum parameter variations (listed in Table 3).

**Table 1:** FBR initial conditions.

Bed Initial Conditions			
Temperature (Gas & Solids)		650 °C	
N_2_ mass fraction		0.222	
H_2_O mass fraction		0.691	
CO_2_ mass fraction		0.087	
Average Bed Height		65 in.	
Bed Coal Char Fraction		0.93	
Bed Coal Ash Fraction		0.07	
Bed Material	Composition (wt%)	Skeletal Density (kg/m^3^)	D_p_ (µm)
Product Particles	90	2530	275
Coal	10	1092	500

**Table 2:** Base parameter conditions.

Flow to Ring and Rails	
Ring Flow Rate (H_2_O)	174.11 kg/h
Rail Flow Rate (H_2_O/O_2_)	257.87 kg/h
Temperature	530 °C
Injection Feed (Total)	
Temperature	21 °C
Flow Rate	0.363 m^3^/h
Droplet Size	75 µm
H_2_O mass fraction	0.6234
Solids/aqueous mass fraction	0.3766
Purge from bottom of FBR	
Flow Rate	174.52 kg/h
N_2_	100%
Temperature	200 °C
Atomizing Gas (Total)	
Temperature	21 °C
Flow Rate (Air)	140 kg/h

**Table 3:** Parameter variations for LHS.

Parameter	Low Value	Base Value	High Value
Bed Particle Size [μm]	200	275	350
Spray Injection Flow Rate [m^3^/h] (2 nozzle)	0.114	0.182	0.284
Bed Particle Density [kg/m^3^]	2200	2530	2650
Coal Particle Size [μm]	50	500	1000
Fluidizing Gas Flow Rate [kg/h]	259	432	605

**Fig. 2:**
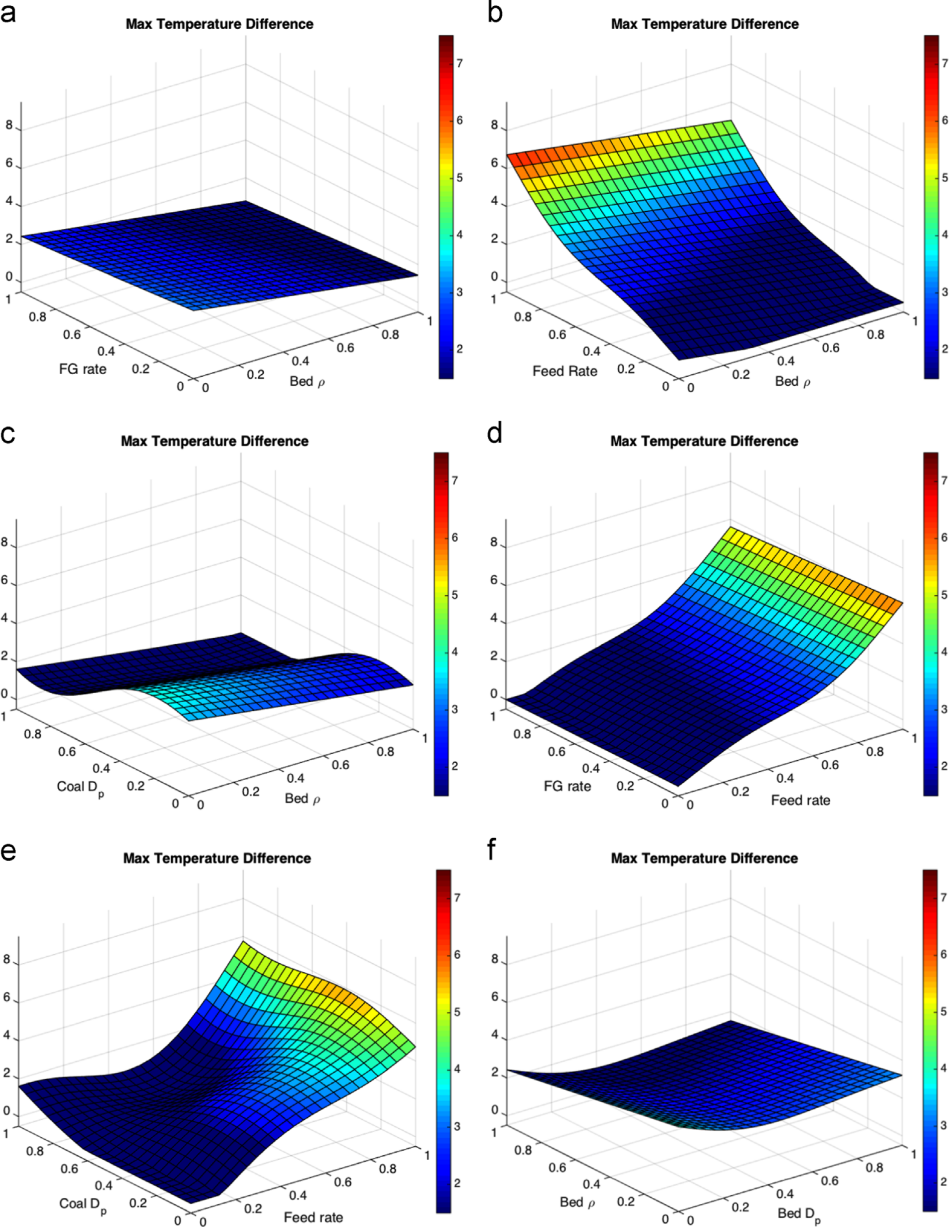

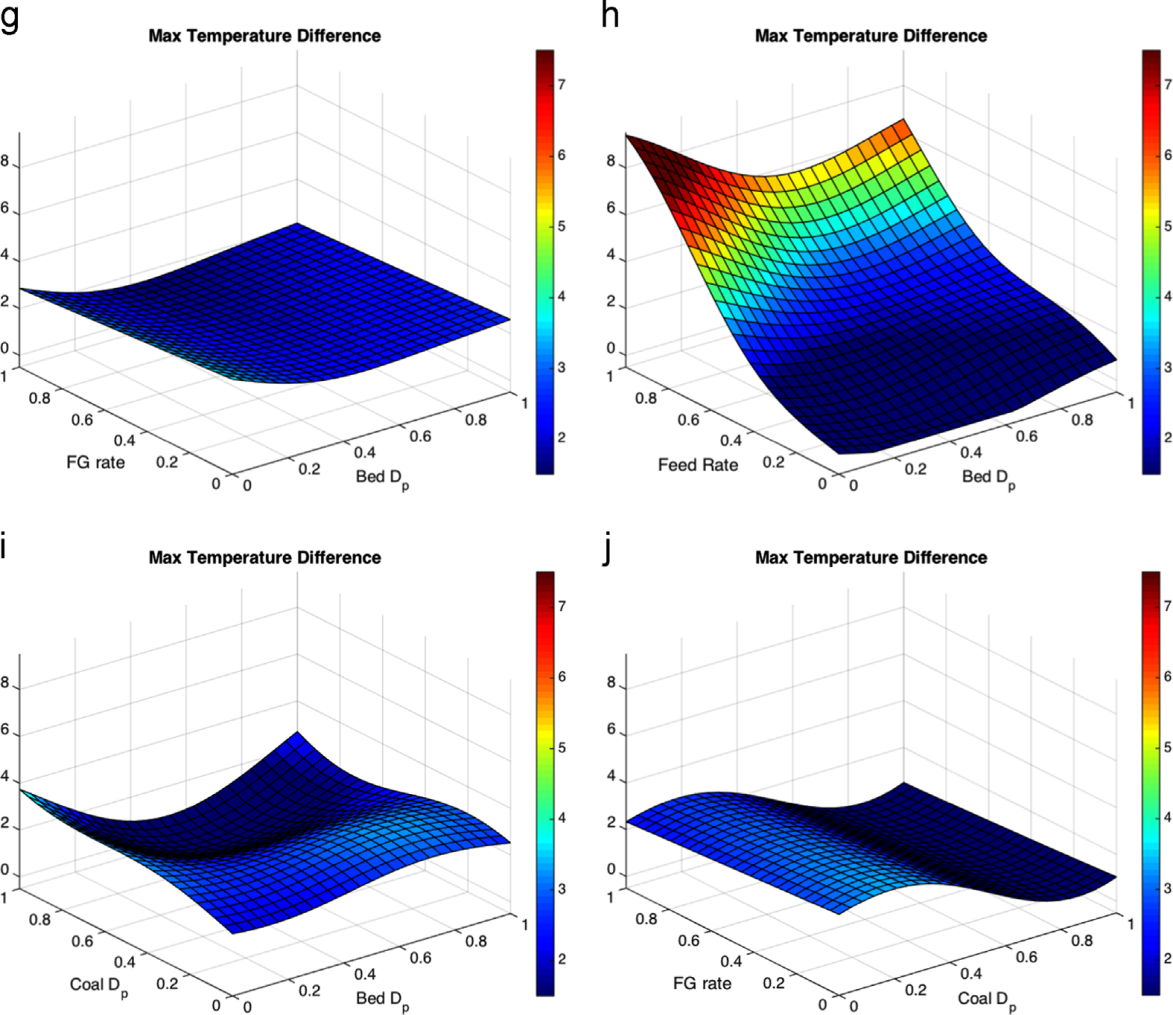
Response surfaces for temperature differential as a function of (a) fluidizing gas flow rate and bed particle density, (b) feed flow rate and bed particle density (c) coal particle size and bed particle density, (d) fluidizing gas flow rate and feed flow rate, (e) coal particle size and feed flow rate, (f) bed particle density and bed particle size, (g) fluidizing gas flow rate and bed particle density, (h) feed flow rate and bed particle size, coal particle size and bed particle size and (j) fluidizing gas flow rate and coal particle size.

**Fig. 3:**
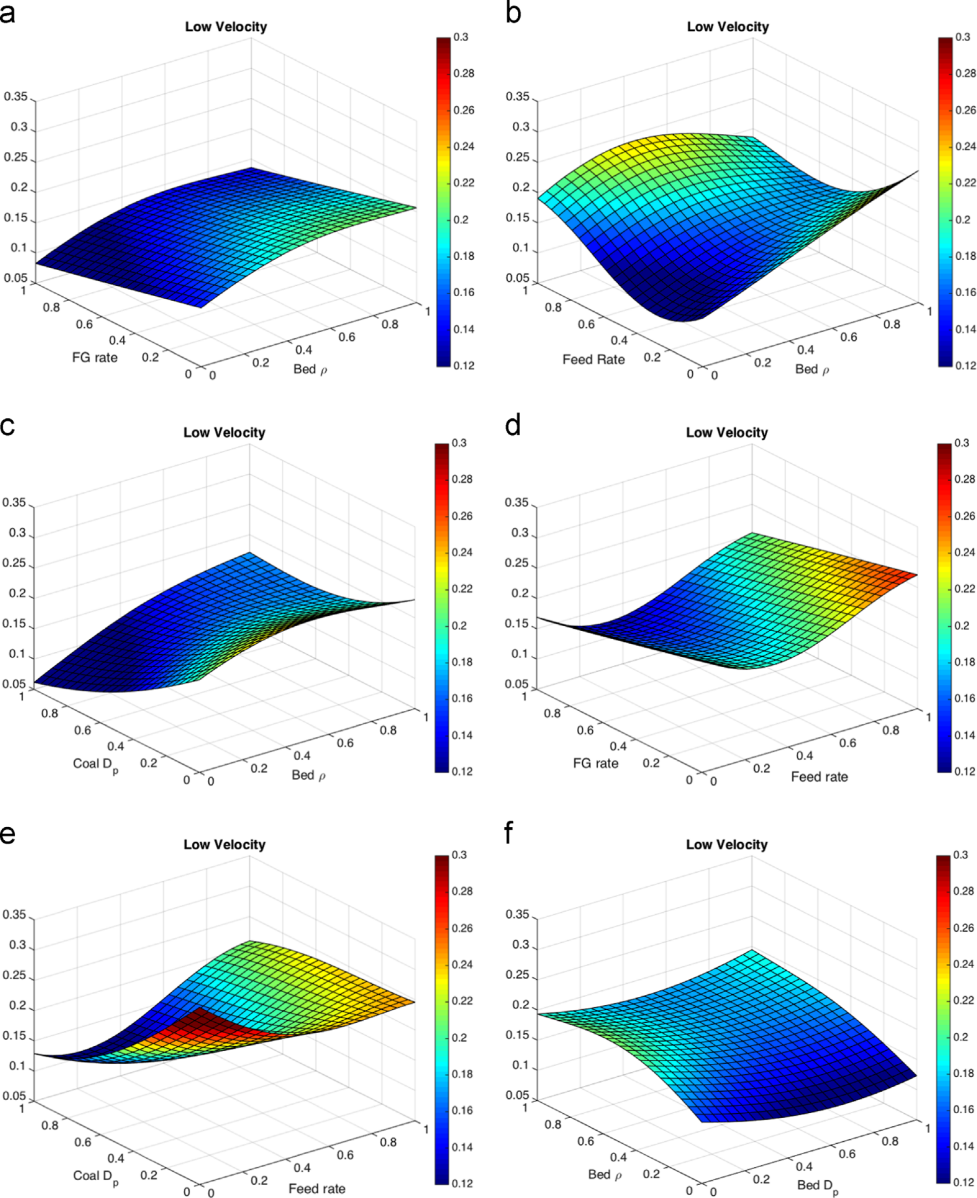

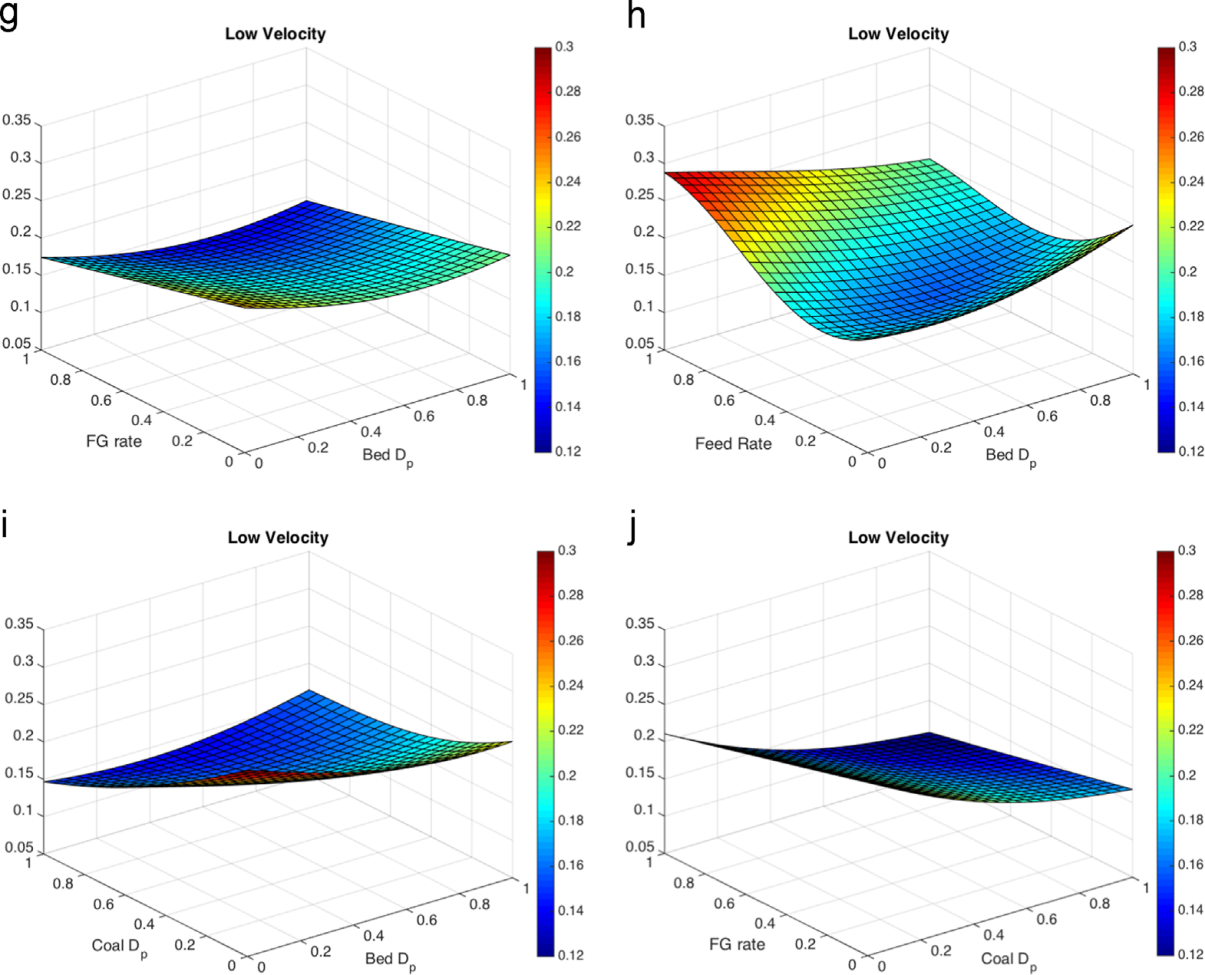
Response surfaces for low solids velocity threshold for (a) fluidizing gas flow rate and bed particle density, (b)feed flow rate and bed particle density (c)coal particle size and bed particle density, (d) fluidizing gas flow rate and feed flow rate, (e) coal particle size and feed flow rate, (f) bed particle density and bed particle size, (g) fluidizing gas flow rate and bed particle density, (h) feed flow rate and bed particle size, (i) coal particle size and bed particle size and (j) fluidizing gas flow rate and coal particle size.

**Fig. 4:**
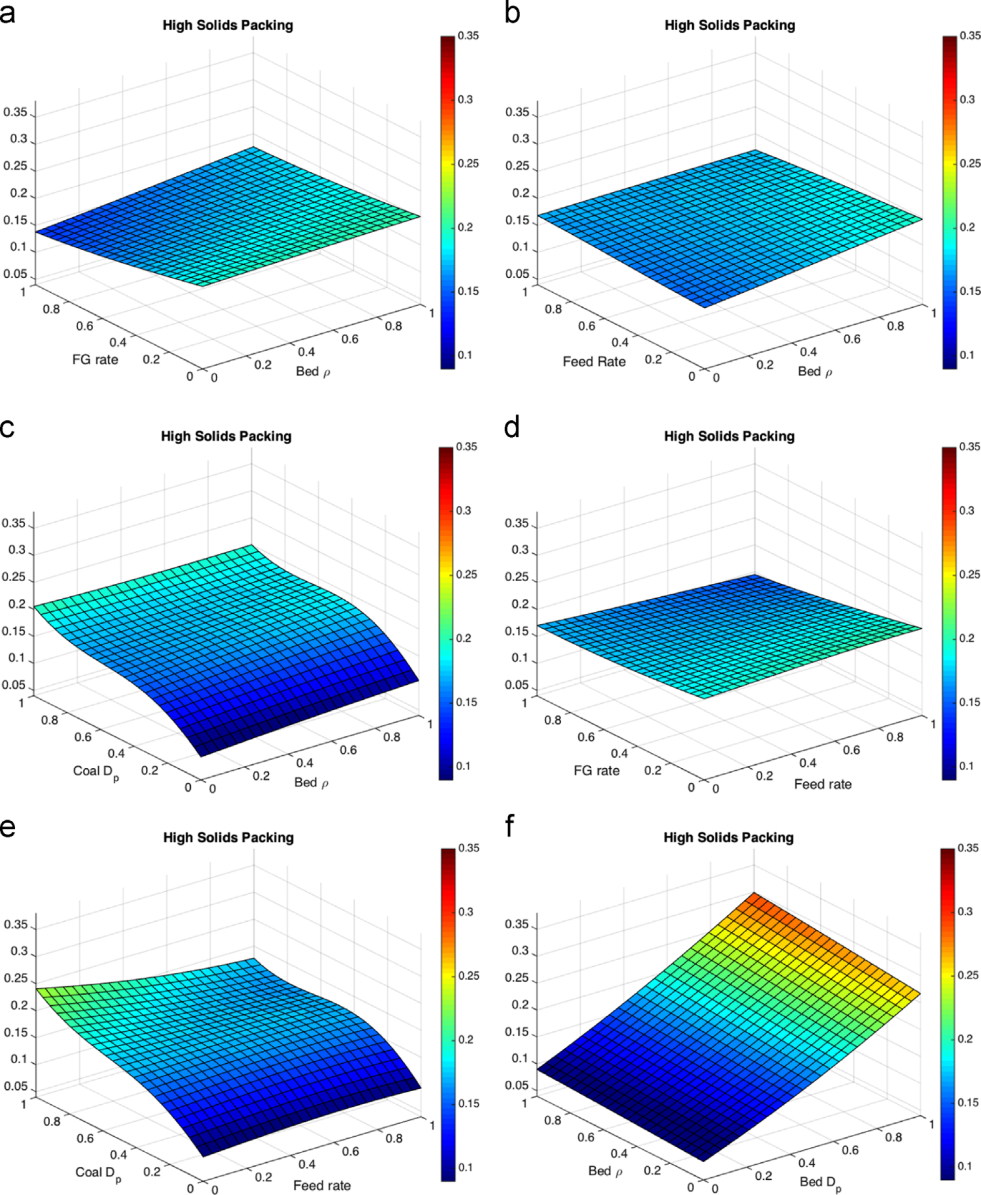

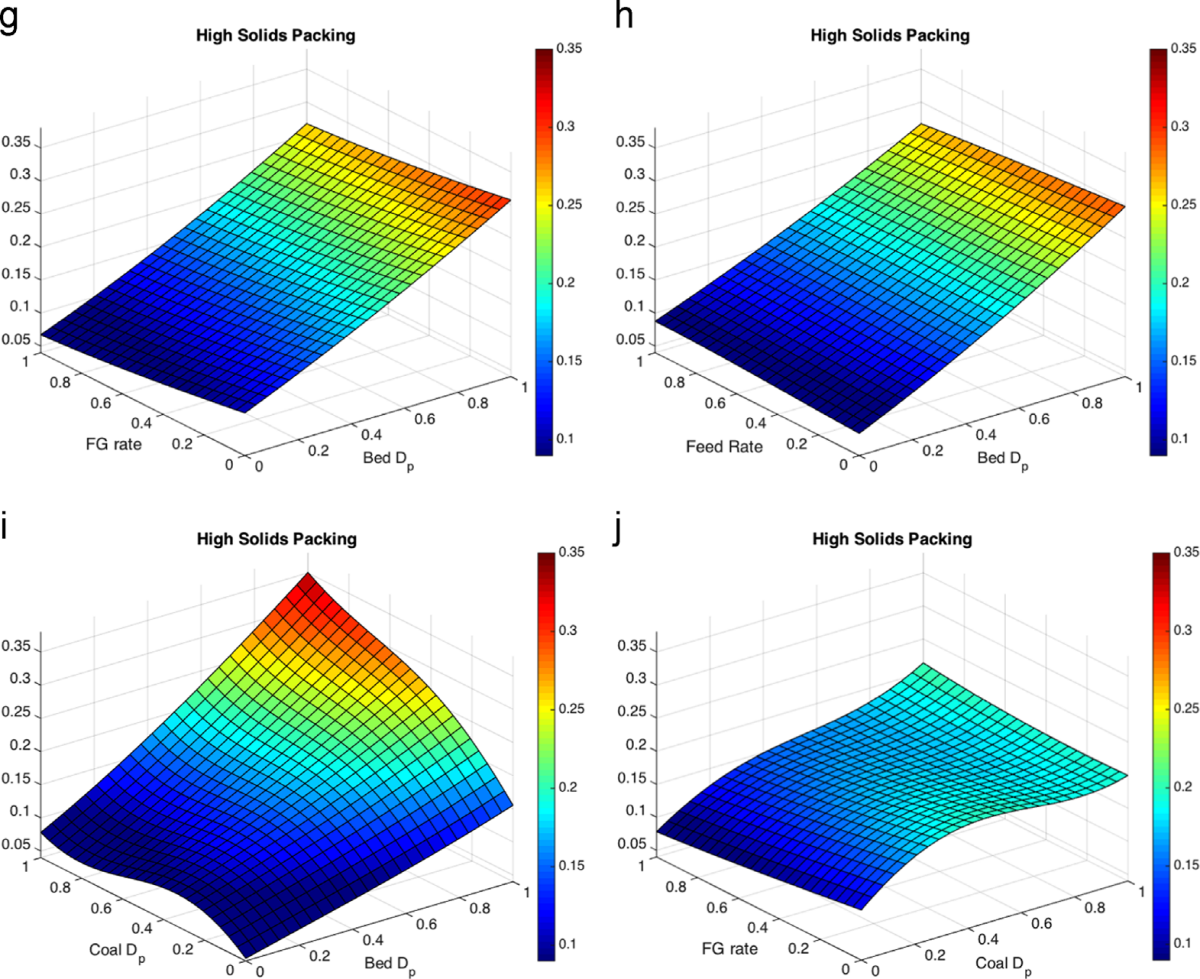
Response surfaces for high solids packing for (a) fluidizing gas flow rate and bed particle density, (b) feed flow rate and bed particle density (c) coal particle size and bed particle density, (d) fluidizing gas flow rate and feed flow rate, (e) coal particle size and feed flow rate, (f) bed particle density and bed particle size, (g) fluidizing gas flow rate and bed particle density, (h) feed flow rate and bed particle size, coal particle size and bed particle size and (j) fluidizing gas flow rate and coal particle size.

When processing the simulation data, the threshold for the FBR considered to be at a low solids velocity was set to a magnitude of 0.3 m/s. The threshold of the FBR considered to have a high solids packing was determined by a void fraction below 0.5. For the temperature differential the point temperatures of approximate thermowell locations were averaged in one-second intervals, then the maximum was found and reported. The results of the five parameter response surface consist of 10 figures, each shown at the average value (0.5) of the other three parameters. The response surfaces for the temperature differential are shown in Fig. 2. The response surfaces for the low solids velocity threshold are shown in Fig. 3. The response surfaces for the high solids packing (low void fraction) are shown in Fig. 4.

Most literature results limit finds of the variation of a small number of parameters without cross-correlating effects such that a direct comparison is hard to make here, but general trends appear to be consistent. The maximum temperature distance shows the liquid feed rate as a large effect in the response surface, which is expected to drive up temperature drops in fluid coking systems [13]. Coal particle size has a moderate impact on the temperatures from the volatile gases. The liquid feed rate is expected to have little impact on the void fraction below the nozzle measured with gamma-ray transmission [14], and little effect on the velocity below the nozzle [15], consistent with the results shown here. Within the ranges in the study, bed and coal particle sizes have the most impact on void fraction and low particle velocity. Fluidization issues based on particle sizes have been a well-documented as an area of concern in fluidized beds [16], [17].

The Gaussian process regression surfaces used to create these models have an underlying linear curve. The parameters for these model fits are listed in Table 4. These coefficients are used in Eqs. (1), (2), (3), (4) to reproduce the response surface plots, where x is the vector of all five input parameters.

(1) $$g\left(x\right)=Y_{mult}h\left(x_{s}\right)+Y_{mult}r{\left(x_{s}\right)}^{T}m+Y_{shift}$$

with

(2) $$h\left(x\right)=\sum_{k}c_{k}\prod_{i}x{\left(i\right)}^{p\left(k,i\right)}$$

and

(3) $$r\left(x;j\right)=\exp\left(-\sum_{i}corr\left(i\right){\left(x\left(i\right)-x_{build}\left(i,j\right)\right)}^{2}\right)\;$$

**Table 4:** Gaussian process parameters.

Coefficient	Low Velocity	Low Void Fraction	Temperature Difference
$Y_{\mathrm{shift}}$	0.2174	0.2182	4.6542
$Y_{\mathrm{mult}}$	0.1855	0.2747	7.5903
$x_{\mathrm{shift}}$	[ 0.5, 0.5, 0.5, 0.5, 0.5]		
$X_{\mathrm{mult}}$	[1.0, 1.0, 1.0, 1.0, 1.0]		
c	[0.1632,0.0615,0.4857, -0.7648, -0.6673, -0.3695]	[-0.0414,0.7227,0.0038, -0.1681,1.0128, 0.0377]	[-0.1207, -0.0904, -0.2121, 0.7959, 0.4010, -0.0701]
corr	[0.3912,0.8449,1.2961, 0.6536,0.0276]	[0.1973,0.0838,0.2341, 1.1898, 0.1401]	[2.7998,0.0300,3.9426, 2.3594, 0.0276]
p	[0 0 0 0 0;		
	1 0 0 0 0;		
	0 1 0 0 0;		
	0 0 1 0 0;		
	0 0 0 1 0;		
	0 0 0 0 1]		
m	[0.4909, -0.2400, -3.2420, -2.4279, 2.4065, 0.1199, 1.9911, 0.5975, -2.7899, 3.7040, -1.7914, 0.7397, 1.2943, -5.7550, -1.0200, 0.0665,3.6765,4.1957, -0.5590, -2.0294,1.5537]	[-2.7378,1.4264,13.7529, 4.2330, -2.6906, -1.1923, 9.0136, -0.6552, -10.8803, -1.8675, -4.2533, -3.7350, 6.6053, -6.2010, -0.3273, 0.6164, -0.9596, -0.4295, 0.2428, 3.1266, -3.0877]	[-0.7387,0.3930, 1.7592, -0.1306, -0.3419, -0.1267, 1.1657,0.4170, -1.0717, -0.7691, -0.5798, -0.0109, -0.1119,0.0485, -0.0185,0.2977,0.8291,0.0508,0.1708,0.2783, -1.5102]
$x_{\mathrm{build}}$	[-0.2701,0.4723,0.1207, -0.2470, -0.0171, -0.3493,0.4116,0.1922,0.0703, 0.3735, -0.0805, 0.2290, -0.3710,0.0804, -0.0339, -0.4182, -0.4914, -0.1203,0.3230, -0.2009,0.2838;		
	-0.1977, -0.3406,0.0077, -0.3996, -0.2955, -0.0499,0.2430, -0.1492,0.1437, -0.4519,0.3546,0.2626, -0.2600, -0.4645,0.1879,0.0267, -0.0793, 0.1170, 0.4360, 0.4691, 0.3627;		
	-0.0328,0.4599, -0.1283,0.4128,0.2260, -0.1047, -0.4246,0.1360, -0.3922, -0.3403,0.3181,0.2891,0.0205, -0.2236, -0.1948, -0.4919,0.3641, -0.2823, 0.1736, 0.1040, 0.0712;		
	0.0871, -0.1402, -0.0102, 0.4142,0.2296, 0.1213, 0.3738, -0.0729,0.2673, -0.1710, -0.3707,0.4773, -0.3257, -0.2180, -0.4314,0.3304,0.0439, -0.4533, -0.3071, -0.0426, 0.1695;		
	0.4372, -0.0496, -0.4544, -0.1421,0.4672, -0.4299,0.2672,0.2167, -0.2600, -0.0715, -0.1815, 0.1386,0.1751,0.0525, 0.4014,0.0070, -0.2971, -0.3140,0.1161,0.3479, -0.3690]		

Using the normalized value for $x_{s}$

(4) $$x_{s}=(x-X_{shift})/X_{mult}$$

**Fig. 5:**
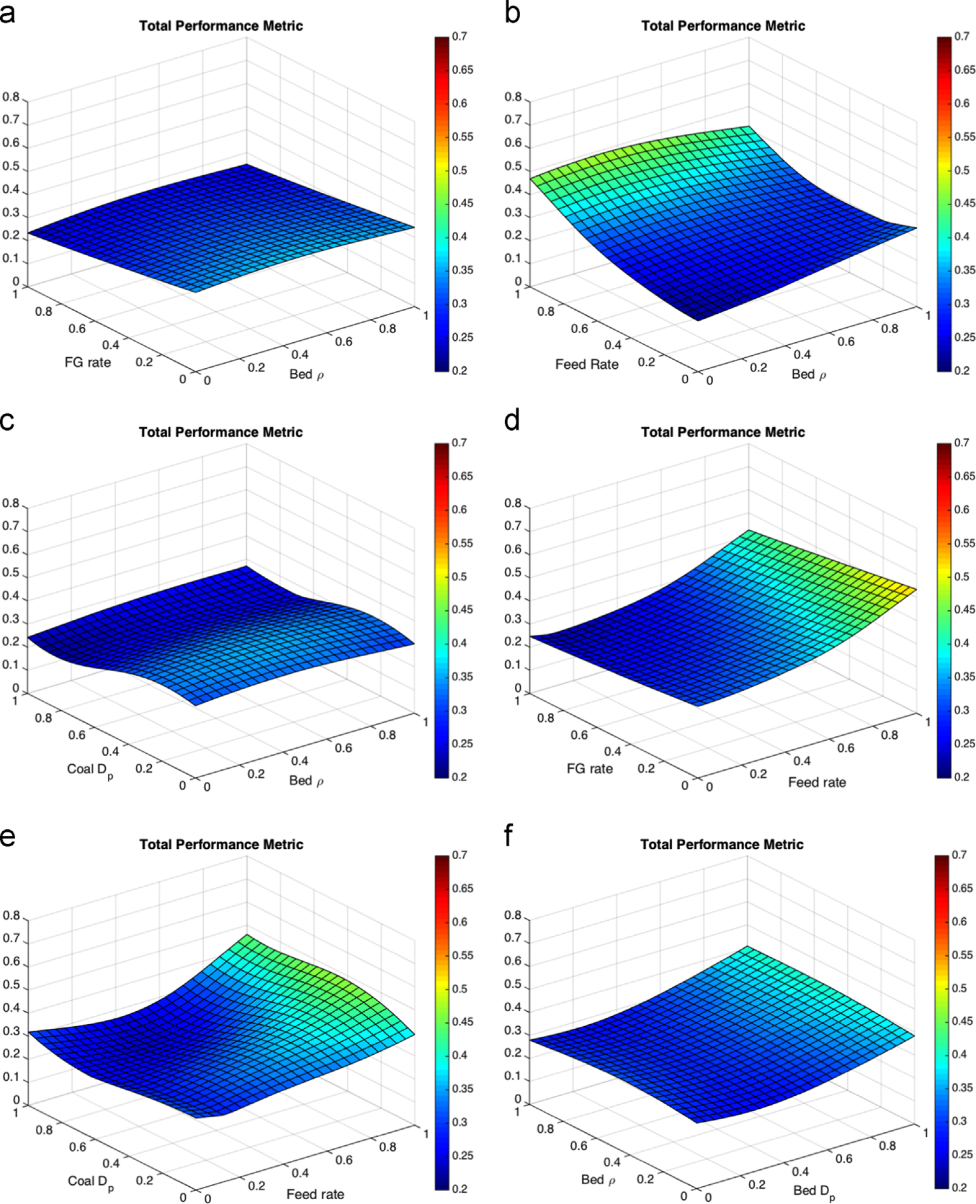

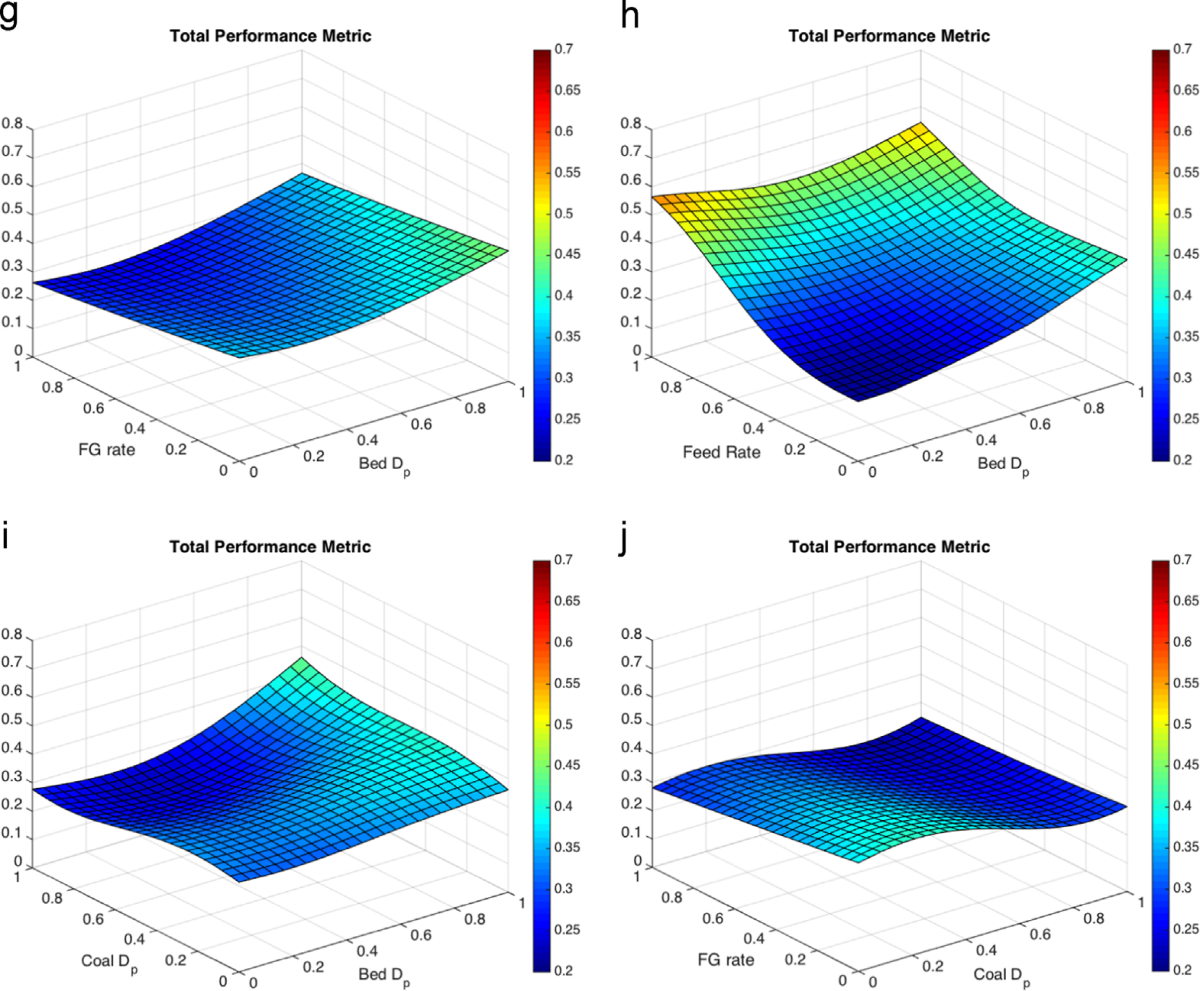
Response surfaces for the total performance metric (a) fluidizing gas flow rate and bed particle density, (b) feed flow rate and bed particle density (c)coal particle size and bed particle density, (d) fluidizing gas flow rate and feed flow rate, (e) coal particle size and feed flow rate, (f) bed particle density and bed particle size, (g) fluidizing gas flow rate and bed particle density, (h) feed flow rate and bed particle size, coal particle size and bed particle size and (j) fluidizing gas flow rate and coal particle size.

A total performance metric surface was created by normalized the three QOIs to the minimum and maximum values. The normalized surfaces were then added to obtain a response surface equally weighting the three QOIs. The response surfaces for the total performance metric are shown in Fig. 5.
